# Review of African swine fever outbreaks history in South Africa: From 1926 to 2018

**DOI:** 10.4102/ojvr.v88i1.1919

**Published:** 2021-09-29

**Authors:** Ciza A. Mushagalusa, Eric Etter, Mary-Louise Penrith

**Affiliations:** 1 Department of Animal Production, Faculty of Agriculture, Université Evangélique en Afrique, Bukavu, the Democratic Republic of the Congo; 2 Department of Production Animals Studies, Faculty of Veterinary Sciences, University of Pretoria, Pretoria, South Africa; 3 UMR ASTRE (Animals, health, Territories, Risks, Ecosystems), CIRAD, Petit-Bourg, France; 4 Department of Veterinary Tropical Diseases, Faculty of Veterinary Sciences, University of Pretoria, Pretoria, South Africa

**Keywords:** African swine fever, distribution, history, outbreak, transmission, domestic pig

## Abstract

The article reviews the outbreaks and distribution of African swine fever (ASF) in South Africa since the first probable outbreak that occurred in the Koedoesrand Ward in 1926. Retrospective data on the ASF outbreaks in South Africa were obtained from the World Organisation for Animal Health (OIE) disease database and the South African veterinary services annual reports in addition to published articles and online sources. South Africa has experienced many outbreaks that can be divided into 2 time periods: the period before the development of the OIE diseases database (1993) and the period after. More than 141 outbreaks of ASF were reported during the first period. Since the development of OIE disease database, 72 outbreaks directly involving 2968 cases, 2187 dead and 2358 killed pigs mainly in smallholder pig farms were reported. The median number of cases for a given ASF outbreak is 17, but in 50% of outbreaks no pigs were killed for prevention. The most important ASF outbreak was reported in April 2014 in the Greater Zeerust district (North West province) involving 326 cases and 1462 killed pigs. However, the outbreak with highest mortality involving 250 pigs was reported in 2016 (Free State province). According to phylogenetic analysis, nine p72 genotypes (I, III, IV, VII, VIII, XIX, XX, XXI and XXII) have been identified in South Africa. Season-wise, more outbreaks were recorded during summer. It was also observed that the OIE disease database could contain errors that would have been introduced through compiled forms at country level. Spatiotemporal studies on ASF outbreaks in South Africa are therefore required in order to assess statistically and quantitatively the clustering of outbreaks over space and time.

## Introduction

In Africa, livestock contributes considerably to the national agricultural economy. According to the Department of Agriculture, Forestry and Fisheries (DAFF) (2012) report, in South Africa livestock constitutes about half of the agricultural output (48%), and represents the vast majority of meat requirement (85%), with the pork industry representing specifically 2.15% of the primary agriculture sector (Boettiger 2000; Munzhelele et al. 2016).

Despite increases in livestock numbers in Africa, the deficit between the supply and demand for animal products remains large because it requires changes in production systems, especially the development of cleanliness and good hygiene that are difficult to maintain as livestock numbers increase (Madzimure et al. 2015). In this situation, animal overcrowding can compromise the immune system through the stress it causes and furthermore facilitates the transmission of infectious diseases (Madzimure et al. 2015). Consequences can be catastrophic, and the result is a strong imperative to provide proof that animals and their products are safe and comply with international trade standards to avoid spread of emerging and transboundary animal diseases (De Klerk 2012). Intensification of production systems that will result from the increased demand for livestock products as well as the impending impacts of climate change have evoked concern worldwide. Notably because of more than 600 million smallholder-farmers worldwide whose livelihoods depend directly on their livestock production (Thornton et al. 2006).

In South Africa, the dualism of the agricultural sector between a sophisticated commercial sector and a subsistence sector represented by small-scale production with low capital investment has created an imbalanced distribution of resources and support services (Antwi & Seahlodi 2011; Madzimure et al. 2013). In 2010–2011, the South African pig population was approximately 125 000 sows, mostly being held in the commercial sector (approximately 100 000 sows), but with a significant part (25 000 sows) held in small-scale farms (Mokoele et al. 2014). This dualism is determinant in the spread of animal diseases, as early disease detection and the enhanced biosecurity needed to control them will be difficult in small-scale sector because of lack of resources. It has been found that all African swine fever (ASF) outbreaks reported in South African domestic pigs during the period covered by this study occurred in smallholder pig farms or at least in relatively small enterprises with poor management and low biosecurity (Mokoele et al. 2014). African swine fever is a fatal, haemorrhagic, highly contagious viral disease with serious global implications, caused by a large cytoplasmic double-stranded deoxyribonucleic acid (DNA) arbovirus, named the African swine fever virus (ASFV), belonging to the genus *Asfivirus*, family *Asfarviridae* (Alonso et al. 2018).

In 1935, the northern parts of the Limpopo, Mpumalanga, North West and KwaZulu-Natal provinces were proclaimed as an ASF Control Area on the basis of the distribution of the ASF epidemiological sylvatic cycle. So far 24 different genotypes of this virus have been described to be circulating in Africa, and 9 genotypes have been identified in South Africa based on the p72 sequencing (Achenbach et al. 2017; Bastos et al. 2003; Boshoff et al. 2007; Quembo et al. 2018). Because of its case fatality rate approaching 100% for peracute and acute forms of the disease, its transboundary nature, the trade restrictions in affected regions and the fear it instigates in affected communities, many authors consider it as the most dangerous swine disease (Chenais et al. 2017; Costard et al. 2009; Hess 1971; Penrith & Vosloo 2009; Plowright et al. 1994; Vial et al. 2007).

The most devastating effects are observed among small scale or poorer pig producers. Constrained with financial and resource limitations, they are not able to ensure basic biosecurity or appropriate prevention and control measures (Edelsten & Chinombo 1995). Because of the lack of compensation schemes, restarting production activities after outbreaks is often difficult and this can consequently lead to the loss of the pig population, which can impact negatively on food security (Costard et al. 2009).

In order to examine the context in which ASF outbreaks occur in domestic pigs, reviewing ASF outbreak history and clustering their distribution is a priority as it has been found that prevention programmes’ effectiveness varies over time and space. To achieve identification of ASF patterns, the knowledge of its history, epidemiology, and the identification of risk factors for disease occurrence are essential (Vergne et al. 2016). Mapping disease outbreak distribution represents an important tool for planning health campaigns, surveillance, and interventions, as it facilitates the identification of transmission high-risk areas and orientates control activities (Mott et al. 1995). Therefore, this study aimed to explore ASF outbreaks history in South Africa in order to develop appropriate control measures and prevention strategies.

## Method

### Data collection

Retrospective data on ASF outbreaks were obtained from the OIE disease database (from October 1993 to May 2018), OIE reports, reviewed published articles, online sources, the veterinary services annual reports (data before 1993), and personal communications with officials involved in outbreaks control and eradication. The World Organisation for Animal Health (OIE) diseases database comprised the year and the month of outbreak, the province, the state veterinary area, the district, the species, the number of outbreaks, cases, dead and killed animals.

### Data analysis

Descriptive analysis was performed on ASF outbreaks in order to describe their spatial and temporal (seasonal) distribution. Seasonal comparison was done using a chi-square test with a significance level of *p* < 0.05.

### Ethical considerations

This article followed all ethical standards for research without direct contact with human or animal subjects.

## Results

### Distribution of warthogs and *Ornithodoros* ticks

In 1935, the northern parts of the Limpopo, Mpumalanga, North West and KwaZulu-Natal provinces were proclaimed an ASF Control Area on the basis of the distribution of the ASF epidemiological sylvatic cycle. This sylvatic cycle is defined by ASFV transmission between the common warthog (*Phacochoerus africanus*) and burrow-inhabiting ticks of the *Ornithodoros moubata* complex (Ixodoidea: Argasidae) during ticks’ feeding and the repeated ASF outbreaks in domestic pigs (Chenais et al. 2017; Penrith & Vosloo 2009; Sánchez-Vizcaíno et al. 2015). Ticks are infected by ASFV during the short viraemic phase of infection in warthog piglets, while the transmission between ticks may be sexual, transovarial and transstadial (Jori et al. 2013; Plowright et al. 1970; Thomson 1985). Despite long-term persistence, ASFV is genetically stable in host ticks, which are the intermediate vector between warthog and domestic pigs or other warthogs (Plowright et al. 1970).

### Distribution of African swine fever virus genes in South Africa

In South Africa, Bastos et al. (2003) and Boshoff et al. (2007) identified seven p72 genotypes out of the 24 genotypes recovered in Africa clustered in three lineages (Table 1). Genotypes III and VII, which have a transboundary distribution (northern South Africa and neighbouring Botswana) and IV, XIX, XX, XXI and XXII, are found in several regions in Africa. Phylogenetic analyses have shown links between virus variants that have caused epidemics that are not related in time (SPEC/53, SPEC/120, SPEC/125, SPEC/251 and RSA/2/96) and differentiated between viruses previously thought to be part of a single outbreak (example: RSA/1/95 and RSA/5/95), describing the genotype-richness of ASFV in South Africa following the designation used by Bastos et al. (2003), Lubisi et al. (2005) and Boshoff et al. (2007). In addition, two more p72 genotypes (I and VIII) of ASFV were isolated from sylvatic hosts (ticks) in the Kruger National Park (KNP). The genotype I was isolated in 1978 and 1981 (Zsak et al. 2005); while the genotype VIII was isolated in 2001 (Janse van Rensburg et al. 2020).

**TABLE 1:** Distribution of the African swine fever viruses and genes characterised in South Africa.

Virus name	Geographical origin	Sampling year	p72 Genbank accession no.	p72 genotype	CVR Genbank accession no.
Lillie	South Africa	1973	DQ250109	XX	DQ250086
24823	Pietersburg	1975	DQ250110	XX	DQ250087
SPEC/53	Letaba	1985	DQ250111	XXI	DQ250088
SPEC/120	Potgietersrus	1987	AF302812	XIX	DQ250089
SPEC/125	Ellisras	1987	DQ250112	XIX	DQ250090
SPEC/245	Louis Trichardt	1992	DQ250117	XXII	DQ250095
SPEC/251	Rustenburg	1992	DQ250118	XIX	DQ250096
SPEC/257	Ellisras	1993	DQ250120	III	DQ250098
SPEC/260	Thabazimbi	1993	DQ250121	VII	DQ250099
RSA/1/95	Hoedspruit	1995	DQ250123	XX	DQ250101
RSA/5/95	Ellisras	1995	DQ250124	III	DQ250102
RSA/1/96	Gravelotte	1996	DQ250125	XXI	DQ250103
RSA/2/96	Pienaarsrivier	1996	DQ250126	XIX	DQ250104
RSA/3/96	Pienaarsrivier	1996	DQ250127	XIX	DQ250105
RSA/1/98	Potgietersrus	1998	AF302818	VII	DQ250106
RSA/1/99Wb	South Africa	1999	AF302818	IV	DQ250108

*Source*: Bastos, A.D.S., Penrith, M.-L., Crucie’re, C., Edrich, J.L., Hutchings, G., Roger, F. et al., 2003, ‘Genotyping field strains of African swine fever virus by partial p72 gene characterization’, *Archives of Virology* 148, 693–706. https://doi.org/10.1007/s00705-002-0946-8 and Boshoff, C.I., Bastos, A.D.S., Gerber, L.J. & Vosloo, W., 2007, ‘Genetic characterization of African swine fever viruses from outbreaks in southern Africa (1973-1999)’, *Veterinary Microbiology* 121(1–2), 45–55. https://doi.org/10.1016/j.vetmic.2006.11.007

ASF, African swine fever; no., number; CVR, Centre for Virus Research.

### Review of previous African swine fever disease outbreaks in South Africa

In the frame of this study, the history of ASF in South Africa is divided into two periods: the period before the development of disease database (OIE data) in 1993 and from October 1993 up to May 2018, period covered by the OIE data.

### African swine fever disease outbreaks in South Africa before 1993

**FIGURE 1:**
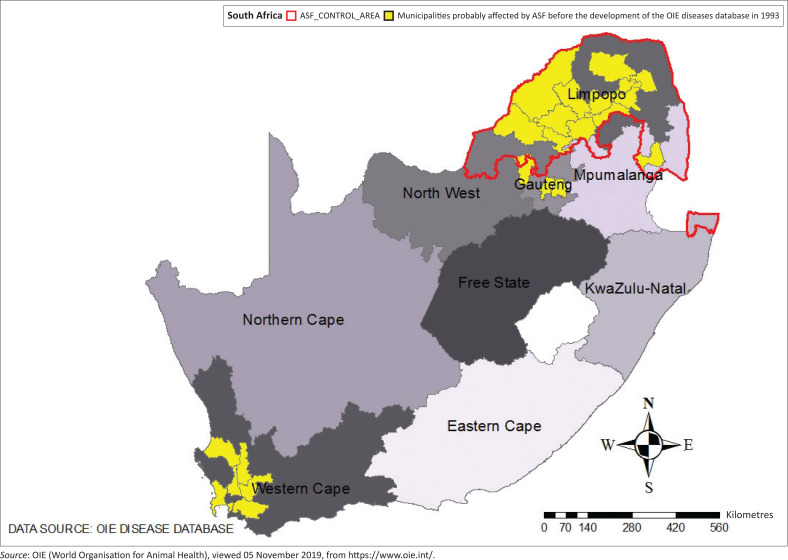
Distribution of probable swine fever in South Africa before the development of the Organisation for Animal Health diseases database in 1993.

Figure 1 illustrates local, metropolitan municipalities and consequently provinces probably affected by African swine fever before the development of the OIE diseases database in 1993.

The Limpopo province in the endemic region was the main region affected by swine fever.

Outbreaks of ‘swine fever’ reported from the Western Cape Province between the turn of the century and 1918 were attributed to classical swine fever (CSF), the only known ‘swine fever’ at that time. This diagnosis was supported by Arnold Theiler’s investigations in 1905 (De Kock, Robinson & Keppel 1940; Penrith 2013). The first ASF outbreak in South Africa was reported in the northern part of the country, Koedoesrand Ward, specifically in the vicinity of Maasstroom, in August 1926 (Steyn 1928).

According to farmers, at that period it was impossible to raise pigs because of that disease, affecting more free roaming pigs of all ages than animals kept in sties, especially on farms with a large number of warthogs in potential proximity to the pigs. In Eastern Europe, it has been shown that wild boar and free-roaming pigs’ direct contact when scavenging and/or inhabiting similar ranges contributes significantly to the spreading of ASF (Melletti & Meijaard 2017). However, the situation involving the sylvatic cycle in Africa differs from that in Europe, because transmission between Eurasian wild boars is direct owing to the fact that they are conspecific with domestic pigs and equally susceptible to the disease, and dead wild boars have proven a potent source of infection (Beltrán-Alcrudo et al. 2017), whereas warthogs do not develop clinical signs of ASF or die of it and transmission to pigs depends on the ticks. (Jori et al. 2013). Steyn (1928) stated that this disease was often a sudden attack and infected animals were characterised by dullness, heavy breathing, grinding of teeth, and foaming in the mouth.

Steyn found that this ‘Koedoesrand disease’ had a number of similarities with the East African swine fever described by Montgomery (1921), such as the incubation period, symptoms and post-mortem lesions, probable association with warthogs, and the immunity developed by affected pigs.

A number of ASF outbreaks, very similar to the disease described by Steyn (1928) and Walker (1933), were reported in the 1930s in Western Cape Province and former Transvaal region (encompassing the current Gauteng, Limpopo, Mpumalanga, the main part of North West and a tiny portion of KwaZulu-Natal provinces). These outbreaks were attributed to pig movements and associated with the presence of warthogs in the north-eastern part of South Africa that was proclaimed as ‘a Swine Fever Control Area’ in 1935. In the control area, strict control measures are applied and pig movements are restricted.

A number of outbreaks occurred in this period, but were mostly recurrences on the same farms. Some of the reported outbreaks, according to De Kock et al. (1940), were:

- Lombardy Estate outbreak, where the owner lost all his 30 pigs, was characterised by high fever followed by death in two to three days;
- Cairngorm Estate outbreak, probably infected by the previous farm but this has never been confirmed;
- Savoy Estate outbreak, in February 1934, when 50 pigs died within a few days most of time 12–24 h after presenting symptoms and sometimes without symptoms. These outbreaks occurred in the Gauteng province.

From the experience gained from previous outbreaks, routine examination was introduced in the Northern Transvaal region. In the year 1934–1935, no outbreak was reported in this region; in 1935–1936, one outbreak was reported in Potgietersrus, two in the Waterberg, and three in Zoutpansberg. In 1937, two outbreaks occurred in the Pietersburg district. Most of the pigs (85%) in this region were not enclosed while up to 25% were not controlled at all and ran free, which facilitated contacts with wild suids reservoirs that resulted in infection (De Kock et al. 1940).

In the Western Cape Province, an outbreak was reported at the Imperial Cold Storage Company, Gouda, Tulbagh district, in pigs destined for slaughter in October 1933. These were believed to have been infected by pigs coming from Johannesburg. The Gouda outbreak was probably the source of outbreaks observed on Onverwacht farm and in Wellington. Towards the end of 1934, another outbreak where about 300 pigs died occurred in Retreat, a suburb of Cape Town. In the year 1934–1935, several outbreaks were observed and the disease became widespread and quarantine measures with prohibition of movements of pigs was introduced in a large part of the country, an area of about 600 square miles. Another outbreak was observed in the Piquetberg area (De Kock et al. 1940).

In 1936, five outbreaks were reported in the Western Cape (two in Wellington, one in Caledon, one in Franschhoek and one in Worcester). The Caledon outbreak extended from Elgin to Villiersdorp. Free-ranging pig movements, the local custom of distributing pork products and the sharing of production tools were the key contributing drivers to the spreading of the disease (De Kock et al. 1940; Penrith 2013). In order to control its spread, buffer zones were created that enabled the identification of many unsuspected cases (De Kock et al. 1940).

In 1939, two linked outbreaks were observed in the Western Cape Province, Piquetberg district. The first occurred in a farm where swine fever was previously reported in 1934, but it was probably sourced from another farm where an outbreak occurred a few months earlier. In this latter farm, it was suggested that carriers probably existed and one of them had been sent to the first farm a few months previously (De Kock et al. 1940). Eblé et al. (2019) confirmed that clinically healthy pigs experimentally infected with a moderately virulent virus may be a source of new acute infections in a proportion of exposed pigs up to 55 days post infection, but there is little evidence that such pigs contribute to maintenance of the ASF virus in domestic pig populations (Petrov et al. 2018; Ståhl et al. 2019).

From 1933 up to December 1934. about 11 000 pigs were affected, of which 72.7% were lost. Pigs were often preventively slaughtered before many had died, so that in 82 outbreaks there were only about 328 direct deaths from the disease itself. From December 1934 to June 1935, about 3.610 pigs were destroyed. Although disease outbreaks reported between 1933 and 1939 were confirmed as being swine fever, but were not clearly defined as classical African swine fever. In fact at that time most people, including De Kock et al. (1940), thought that the virus that caused ASF was likely to be a particularly virulent CSF virus. They were closely related to the ‘East African swine fever’ described by Montgomery in 1921, and an association with warthogs in the north-eastern part of South Africa was apparent in the ASF control area (De Kock et al. 1940; Penrith 2013). African swine fever outbreaks were confirmed since 1973 as stated by Bastos et al. (2003) and Boshoff et al. (2007).

In the 1950s. three ASF outbreaks were reported mainly in the Northern Transvaal (Northern Province, now Limpopo) between 1953 and 1956. A dead pig near a farmhouse was suspected of being the source of infection, but annual reports did not give precision of the district or their exact location.

In the 1960s, only one outbreak was reported. In this outbreak, 23 pigs were found infected with ASFV and 49 pigs were slaughtered as a preventive measure. It occurred in 1961–1962 in the district of Soutpansberg (in the current Limpopo province).

In the 1970s, 26 outbreaks were reported in the districts of Giyani, Ritavi, Seshego, Bolobedu, Pietersburg, Letaba, Thabazimbi in the current Limpopo province, White River (in the current Mpumalanga province) and Rustenburg (in the current North West Province) state veterinary areas. Seventeen farms were affected in 1973–1974 after a long period of apparent ASF absence (12 years since 1962). Although it is possible that there were no cases, it is likely that some people would not have reported the loss of a small number of pigs.

In the 1980s, 7 outbreaks were reported in the Transvaal province (concentrated in the current Limpopo province) region.

One outbreak occurred respectively in each of the following: the former Transvaal (1981–1982), but the exact district of occurrence was not determined by the annual report of veterinary services, Letaba district (1985–1986), Waterberg (1985–1986), Potgietersrus district (1986–1987), Nylstroom (1988–1989) and two outbreaks in the district of Ellisras (1989–1990). In the 1990s, just before the development of the OIE disease database in 1993, two outbreaks occurred in Thabazimbi (1992–1993) and Soutpansberg (1992–1993) districts (both in the current Limpopo province).

Rapid detection, determination of the extent of contamination and elimination or disinfection of contaminated pigs and contact herds with payment of compensation were the measures applied to control outbreaks in South Africa. All probable ASF outbreaks reported before the OIE diseases database are presented in Table 2.

**TABLE 2:** Probable African swine fever outbreaks reported before the development of the World Organisation for Animal Health diseases database in 1993.

Year	Number of outbreaks	Regions of occurrence	Provinces (as established in 1994)
1926	1	Koedoesrand Ward (Waterberg)	Limpopo
1933–1934	82	Lombardy, Cairngorm Estates, Savoy Estate (Johannesburg)	Gauteng
		Gouda (Tulbagh district), Cape town, Retreat (suburb of Cape Town), Halfmanshof near Gouda, Riebeek West (Malmesbury district), arm Fruit Grove (Wellington), Prince Alfred’s Hamlet (Ceres), Clanwilliam district, Piquetberg-Clanwilliam (Malmesbury district), Piquetberg	Western Cape
1935	6	Waterberg and Zoutpansberg (Mopani)	Limpopo
1936	5	Wellington, Calledon (Overberg), Franschhoek, and Worcester (Cape Winelands)	Western Cape
1937	2	Pietersburg (Capricorn) district	Limpopo
1938	2	Pietersburg (Capricorn) district	Limpopo
1939	2	Piquetberg	Western Cape
1953–1954	1	Northern Transvaal	Northern province
1954–1955	2	Northern Transvaal	Northern province
1955–1956	2	Northern Transvaal	Northern province
1961–1962	1	Soutpansberg (Vhembe district)	Limpopo
1973–1974	26	Giyani, Thabazimbi districts, Seshego and Pietersburg in Capricorn district, Bolobedu and Letaba in Mopani district	Limpopo
		Ritavi district	Gauteng
		White river (Ehlanzeni district)	Mpumalanga
		Rustenburg (Bojanala Platinum district)	North West
1975–1976	2	Lebowa, Potgietersrus district	Limpopo
1976–1977	2	Rustenburg State veterinary (Bojanala Platinum)	North West
1977–1978	1	Rustenburg state veterinarian area (Bojanala Platinum)	North West
1978–1979	3	Thabazimbi district	Limpopo
1979–1980	1	Letaba (Mopani district)	Limpopo
1981–1982	1	Transvaal region	
1985–1986	2	Letaba (Mopani district) and Waterberg	Limpopo
1986–1987	1	Potgietersrus district	Limpopo
1988–1989	1	Nylstroom (Modimolle district)	Limpopo
1989–1990	2	Ellisras district	Limpopo
1992–1993	2	Thabazimbi and Soutpansberg districts	Limpopo

*Source:* Adapted from De Kock, G., Robinson, E.M. & Keppel, J.J., 1940, ‘Swine fever in South Africa’, *Ondersterpoort Journal of Veterinary Research* 14, 31–93.

### African swine fever outbreaks distribution in South Africa from 1993 up to May 2018

**FIGURE 2:**
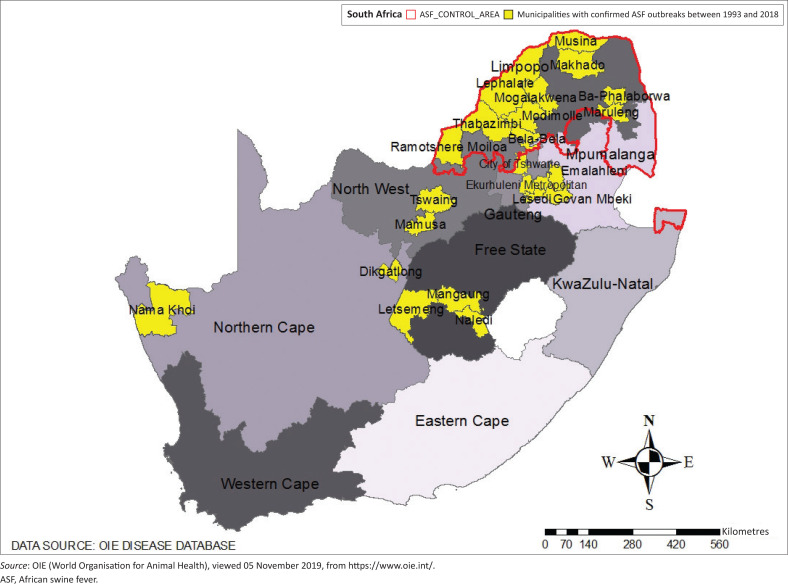
Distribution of municipalities where African swine fever outbreaks have been reported in South Africa from 1993 to 2018.

Since 1993, DAFF has reported laboratory-confirmed ASF outbreaks to the OIE, with their date (year and month), location (district, province), and number of killed pigs, case morbidity and mortality data. In order to preserve the anonymity of the farm, a jamming system is used for GPS coordinates. Figure 2 illustrates local, metropolitan municipalities and provinces where ASF outbreaks have been reported to the OIE from October 1993 to May 2018.

African swine fever outbreaks occurred mainly in Limpopo province, with 35 outbreaks (48.6%), and other northern South African provinces such as Gauteng, Mpumalanga, North West and Free State provinces, representing respectively 8.3 (6 outbreaks), 12.5 (9 outbreaks), 8.3 (6 outbreaks) and 15.8% (11 outbreaks) of outbreaks. In this interval, one outbreak was reported in KNP and four in the Northern Cape Province. It seems that this incidence of ASF in the KNP is a result of the change in the geocoding of the information done by DAFF, as no outbreaks inside the parks are likely because there are no domestic pigs inside them.

Alternatively, this reported outbreak to the OIE in the park maybe an occasional test of a positive killed warthog, as the ASF virus can sometimes be detected in lymph nodes. African swine fever antibodies have been found in warthogs in several regions in Eastern and Southern Africa that coincide with the presence of infected ticks (Jori et al. 2013). In endemic zones, up to 100% of warthogs may carry ASFV antibodies (Beltrán-Alcrudo et al. 2017). All reported ASF outbreaks in South Africa occurred in the following local municipalities: Lephalale (11), Mangaung (8), Delmas (7), Thabazimbi (6), Lesedi (5), Musina (5), Dikgatlong (3), Ba-Phalaborwa (3), Makhado (3), Bela-Bela (3), Ramotshere Moiloa (3), Letsemeng (2), Maruleng (2), Tswaing (2), Naledi Govan Mbeki (1) and Naledi Emalahleni (1), Nama Khoi (1), Modimolle (1), Mogalakwena (1) and Mamusa (1).

Thus, DAFF reported 72 confirmed outbreaks directly involving 2968 cases, 2187 dead and 2358 killed pigs, mainly in smallholder pig farms with low biosecurity practices. In Table 3, errors introduced in compiling forms at country level are presented. What one would expect in an outbreak of ASF, particularly in a non-endemic area, would be that the number of dead pigs would be close or equal to the number of cases but not more, because each dead pig would also represent a case. The number of pigs killed can be any number, usually it is the number of susceptible pigs minus the dead pigs if that is provided, but it could be whatever number the vets decided (depending on the chosen policy) should be removed from the population. The most likely explanation for the discrepancy would be that the person filling in the form put the dead and killed pigs in the wrong columns. Thus, to estimate the total number of cases and dead, we have swapped the values corresponding to these columns.

**TABLE 3:** Errors observed in the African swine fever outbreaks reported to the World Organisation for Animal Health between October 1993 and May 2018.

Date	Province	District	Outbreaks	Cases	Dead	Killed
2012/1	Mpumalanga	Govan Mbeki	1	15	69	245
2012/10	Limpopo	Musina	1	5	12	3
2014/4	North West	Greater Zeerust	1	0	326	1462
2017/6	Limpopo	Makhado	2	140	148	148
2017/8	Limpopo	Lephalale	1	1	77	123

### Summary of African swine fever outbreaks in South Africa between 1993 and 2018

The median number of cases for a given ASF outbreak is 17, while it is 10 and 0 respectively for dead and killed pigs (Table 4). For a given ASF outbreak, about 25% of cases involved fewer than 6 pigs, while about 25% involved more than 42 pigs. In case of dead pigs, there were fewer than 4 in about 25% of cases, while in about 25% more than 34 pigs died. In addition, we observed that in 50% of outbreaks no pig was killed for prevention. According to the OIE database, the most important ASF outbreak in South Africa was reported to the OIE in April 2014 in the North West province, Greater Zeerust district. It involved 326 cases and 1462 pigs were killed, while the ASF outbreaks with the most dead pigs involved 250 pigs in 2016, in the Free State province.

**TABLE 4:** Summary of cases, dead and killed pigs by African swine fever outbreaks in South Africa.

Descriptive statistics	Cases	Dead	Killed
Mean	43	32	34
Standard deviation	66	54	178
1st quantile	6	4	0
Median	17	10	0
3rd quantile	42	34	7
Maximum	326	250	1462

### Seasonal distribution of African swine fever outbreaks in South Africa

**FIGURE 3:**
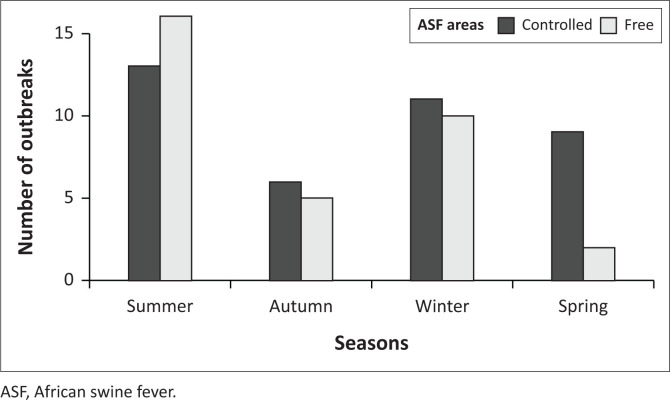
Seasonal distribution of African swine fever outbreaks in South Africa from 1993 to 2018.

The seasonal distribution of outbreaks in South Africa indicated that ASF outbreaks have been reported in all seasons in both the control and free ASF zone as illustrated by Figure 3. The chi-square test has shown a significant difference (*p* = 0.0054) between the proportions of outbreaks in each season. The summer period (December-February) is the season with the highest number of outbreaks, where 29 outbreaks were reported in both controlled and free zones, followed by the winter period (June-August), where 21 outbreaks were reported, and autumn (March-May) and spring (September-November) periods where 11 outbreaks each were reported. Before 2012 and thus within the controlled area there was no seasonal difference.

According to the OIE disease database, before 2012 outbreaks were not reported beyond the demarcation line of ASF control area. Unfortunately, the OIE database used in the frame of this study did not report two spill-overs in the 1990s to farms just outside the red line (Control area limit), in both cases affecting a single farm with few pigs, and easily traced back to the control area (Magadla et al. 2016; Penrith, pers. comm., 2018). One outbreak was linked to a boar that was incubating ASF being brought to a farm just outside the control zone to mate a sow that got sick and died there.

The maximum number of outbreaks for a single year was observed outside the control area, when 15 outbreaks were reported in Mpumalanga (9) and Gauteng (6). We have observed that outbreaks have tended to occur in the free area since 2012 where they were not previously reported.

## Discussion

In order to understand the context in which ASF outbreaks occur in domestic pigs, all outbreaks reported to the OIE from 1993 up to 2018 have been analysed. In addition, all probable ASF outbreaks that occurred in South Africa before the development of the OIE diseases database in 1993 were summarised from 1926 when the first probable cases of African swine fever were recorded. Thus, according to De Kock et al. (1940) and South African veterinary services annual reports from 1954 up to 1993, more than 149 swine fever outbreaks were recorded mainly in the northern part of the country that was then defined as the ‘ASF control area’ but these outbreaks were not clearly defined as classical or African swine fever. This control area was defined according to the local distribution of the infected natural host (Penrith & Vosloo 2009). Surveys of ectoparasites of warthogs in both north-eastern South Africa and Namibia have shown that nymphs of *Ornithodoros moubata* complex ticks can be found on warthogs in fairly large numbers (Boomker et al. 1991; Horak et al. 1983, 1988). Regarding the low probability of direct transmission between warthogs and domestic pigs, the transmission from warthogs to pigs is ascribed to infected ticks transported from the warthog’s burrow to pig’s premises.

Since 1993, DAFF has reported 72 confirmed outbreaks directly involving 2968 cases, 2187 dead and 2358 killed pigs. However, these numbers should be treated with caution as they depend upon the time the outbreak was reported to the OIE and errors can be made in the compiling of the forms at country level. As an illustration, we can consider the outbreak that occurred in Nama Khoi, Northern Cape Province on 23 May 2018 (outside the ASF control area) and resolved on 10 December 2018. According to the OIE disease database, this outbreak affected 14 pigs (14 cases, 14 dead, none killed). This is contrary to what is mentioned in the final report published by the OIE in the frame of the WAHIS (OIE’s web-based platform, World Animal Health Information System) project suggesting that this outbreak was declared resolved on 10 December 2018 and involved 100 animals (susceptible) instead of 14, including 23 cases, 23 deaths and 77 animals killed and disposed of (Modisane 2018). This situation was also observed in reporting the ASF outbreaks considered as the first large scale outbreaks outside of the control area in Ekhurhuleni and Lesedi municipalities that occurred in January 2012. In these outbreaks, the OIE disease database did not report culled animals, while Geertsma, Mpofu and Walters (2012) reported 44 culled animals in Ekhurhuleni and 121 in Lesedi in 5 outbreaks. In addition, the outbreak that was reported by Geertsma et al. (2012) in the Pretoria State Veterinary Area (Schietpoort farm) in Lesedi in January 2012 affecting 5 pigs (5 cases, 3 dead) was not reported to the OIE. Our results showed that the 2012 outbreaks were the first outbreaks reported outside the control area but the official reports did not include the two minor outbreaks in the 1990s involving single farms described above, one of which occurred in 1996 and was mentioned by Magadla et al. (2016). The development of the new OIE-WAHIS should check this discrepancy in order to correct them.

Concerning errors made in the compiling of the forms at country level, 5 errors were observed where the number of dead pigs by ASF was greater that the number of cases, which does not make sense unless the cases represent only the confirmed cases (the ones that were sampled and sent to the laboratory for diagnosis), but this scenario seems less probable than a simple error.

Results obtained in the frame of this work showed that the season has a significant effect on the number of outbreaks despite the fact that they have been reported in all seasons. The summer is the season with the highest number of outbreaks mainly after 2012. It appears logical as more pigs move and are slaughtered around Christmas time, and this seasonal effect is more likely to be linked to pig’s movements rather than climatic and environmental conditions (Penrith & Vosloo 2009). In addition, Arnot, Du Toit and Bastos (2009) suggested that ‘late spring to early summer’ is recognised as the farrowing period for warthogs, with increased viraemia in neonatal warthogs in burrows. It is suspected that some of the spring and early summer outbreaks are related to very dry conditions before the start of the rainy season. Much of Limpopo is fairly marginal climatically, and when warthogs are hungry and thirsty they are more likely to scavenge around pig farms. This would probably not have much of a pattern because the rains are not always late and droughts are fairly irregular, but most likely to occur when the dry season (winter) is prolonged.

These outbreaks occurred mainly in the northern South African provinces (Limpopo, Gauteng, Mpumalanga, North West and Free State) and seem qualitatively clustered in that region despite the fact that since 2012 more and more outbreaks have been reported outside the control area. According to Magadla et al. (2016), other reasons that could explain the clustering of outbreaks could be the distribution of ‘small scale pig farms and socio-cultural and religious practices that influence pig movement and contact with warthogs and infected tampans’. African swine fever outbreaks were reported mostly in relatively small piggeries with conditions of poor management and low biosecurity (Mokoele et al. 2014).

In both prior to and post 1993 periods, commercial and small-scale production systems with low capital have been observed in South Africa (Antwi & Seahlodi 2011). According to Krecek et al. (2008), 25% of pigs in South Africa belong to so-called emerging pig producers or smallholder pig farms. Unfortunately, multiple factors constrain the development and production of smallholder pigs such as disease outbreaks and pig husbandry practices in communal production and free-range systems (Antwi & Seahlodi 2011). In smallholder farms, bad hygienic conditions associated with poor biosecurity encourage disease occurrence and spread (Renaudeau 2009). Their negative effect is accentuated by failure to identify diseases early, high prices of the medication, and lack of experience of extension services, veterinarians and preventive healthcare that result in a high rate of mortality (Tekle, Tesfay & Kifleyohannes 2013). In addition, smallholder farmers utilise risky methods that consist of borrowing boars for breeding from relatives, neighbours, or allow sows to roam freely to be serviced or use boars bought at auction or untested boars (Munzhelele et al. 2016). Feeding of swill from kitchen and restaurant waste have been reported in smallholder pig farming in South Africa and other African countries, which can result in transmission of diseases such as ASF (Mokoele et al. 2014). Considering the increasing numbers of pigs kept, this disease is becoming very important as in the absence of an existing vaccine, the culling of susceptible animals at detection of an outbreak remains the main measure to control ASF outbreaks, and compensation for culled pigs is no longer a policy in South Africa. Thus, better management strategies including improvement of pig husbandry by confined systems and marketing for outbreak prevention become essential (Penrith 2013; Penrith et al. 2013).

## Conclusion

African swine fever remains a serious threat to the pig production sector worldwide. This study explored the history of ASF in South African pig production systems. In South Africa, there is a need to remain aware that ASF control measures should no longer be seen as the sole responsibility of the veterinary services, and that this disease is only a matter for ASF control zone, but a matter for all. All pig producers must take the necessary precautions to ensure that their own herds are not infected. As the success of any disease prevention and/or control programme depends on a better understanding of its epidemiology, further analysis of the available data is needed to clearly understand the distribution patterns of ASF disease. Spatio-temporal studies of ASF in South Africa are therefore needed to statistically and quantitatively assess the clustering of outbreaks in space and time.
